# 苯达莫司汀单药治疗复发/难治B细胞非霍奇金淋巴瘤：一项前瞻、多中心、单臂、Ⅱ期临床研究

**DOI:** 10.3760/cma.j.issn.0253-2727.2022.11.009

**Published:** 2022-11

**Authors:** 岩 高, 瑜 杨, 洪 岑, 红 刘, 晋翔 傅, 顺清 王, 茹 冯, 丁 于, 新友 张, 焯文 陈, 玉富 李, 慧强 黄

**Affiliations:** 1 中山大学肿瘤防治中心肿瘤内科，广州 510060 Department of Medical Oncology, Sun Yat-Sen University Cancer Center, State Key Laboratory of Oncology in South China, and Collaborative Innovation Center of Cancer Medicine,Guangzhou 510060, China; 2 福建省肿瘤医院淋巴瘤及头颈肿瘤内科，福州 350014 Department of Lymphoma and Head and Neck Cancer, Fujian Tumor Hospital, Affiliated Cancer Hospital of Fujian Medical University, Fuzhou 350014, China; 3 广西壮族自治区肿瘤医院肿瘤内科，南宁 530021 Department of Chemotherapy, Guangxi Cancer Hospital and of Guangxi Medical University Affiliated Cancer Hospital, Nanning 530021, China; 4 南通大学附属医院血液科，南通 226001 Department of Hematology, Affiliated Hospital of Nantong University, Nantong 226001, China; 5 苏州大学附属第二医院血液科，苏州 215004 The Second Affiliated Hospital of Soochow University, Suzhou 215004, China; 6 广州市第一人民医院血液科，广州 510000 Department of Hematology, Guangzhou First People's Hospital, School of Medicine, South China University of Technology, Guangzhou 510000, China; 7 南方医科大学南方医院血液科，广州 510515 Department of Hematology, Nanfang Hospital, Southern Medical University, Guangzhou 510515, China; 8 湖北省肿瘤医院肿瘤内科，武汉 430079 Oncology Medicine Department, Hubei Cancer Hospital, Wuhan 430079, China; 9 深圳市人民医院血液内科，深圳 518020 Department of Hematology, The Second Clinical Medical College（Shenzhen People's Hospital）, Jinan University, Shenzhen 518020, China; 10 佛山市第一人民医院血液内分泌科，佛山 528000 Department of Hematology, The First People's Hospital of Foshan, Foshan 528000, China; 11 河南省肿瘤医院血液科，郑州 450008 Department of Hematology, Henan Cancer Hospital, Henan Institute of Hematology, Zhengzhou 450008, China

**Keywords:** 苯达莫司汀, 淋巴瘤，B细胞, 治疗结果, 安全, Bendamustine, Lymphoma, B-cell, Treatment outcome, Safety

## Abstract

**目的:**

评估苯达莫司汀单药治疗中国复发/难治B细胞非霍奇金淋巴瘤（B-NHL）患者的有效性和安全性。

**方法:**

本研究为前瞻性、多中心、开放标签、单臂Ⅱ期临床研究。纳入2012年3月至2016年12月国内11所医院78例接受苯达莫司汀治疗的B-NHL患者，分析患者的临床特征、疗效及生存。

**结果:**

患者的中位年龄为58（24～76）岁，69例（88.4％）患者为Ⅲ/Ⅳ期，61例（78.2％）患者对末线治疗耐药。全组患者中位接受4（1～10）个周期苯达莫司汀治疗。全分析集中，客观缓解率为61.5（95％ *CI* 49.8～72.3）％，完全缓解率为5.1（95％*CI* 1.4～12.6）％。中位随访33.6（95％*CI* 17.4～38.8）个月，中位缓解持续时间为8.3（95％*CI* 5.5～14.0）个月，中位无进展生存（PFS）期和中位总生存（OS）期分别为8.7（95％*CI* 6.7～13.2）个月和25.5个月（95％*CI* 14.2个月～未达到）。最常见的3～4级血液学不良反应为淋巴细胞减少（74.4％）、中性粒细胞减少（52.6％）、白细胞减少（39.7％）、血小板减少（29.5％）和贫血（15.4％）。最常见的非血液学不良反应为恶心（43.6％）、呕吐（33.3％）和厌食（29.5％）。多因素分析显示，苯达莫司汀治疗<4个周期是影响PFS的预后不良因素（*P*＝0.003），既往未接受含氟达拉滨方案是影响OS的预后不良因素（*P*＝0.009）。

**结论:**

苯达莫司汀单药治疗复发难治B-NHL患者有良好的疗效和安全性。

## Abstract (en)

**Objective:**

To evaluate the efficacy and safety of bendamustine monotherapy in Chinese patients with relapsed/refractory（R/R）B cell non-Hodgkin lymphoma（B-NHL）.

**Methods:**

This prospective, multicenter, open label, single-arm, phase Ⅱ study investigated bendamustine's efficacy and safety in Chinese patients with R/R B-NHL. A total of 78 patients with B-NHL in 11 hospitals in China from March 2012 to December 2016 were included, and their clinical characteristics, efficacy, and survival were analyzed.

**Results:**

The median age of all patients was 58（range, 24–76）years old, and 69（88.4％）patients had stage Ⅲ/Ⅳ disease. 61（78.2％）patients were refractory to previous treatments. Patients received a median of 4（range, 1–10）cycles of bendamustine treatment. The overall response rate was 61.5（95％ *CI* 49.8–72.3）％, the median response duration was 8.3（95％ *CI* 5.5–14.0）months, and the complete remission（CR）rate was 5.1（95％ *CI* 1.4–12.6）％. In the full analysis set, median progression-free survival（PFS）and median OS were 8.7（95％*CI* 6.7–13.2）months and 25.5 months（95％*CI* 14.2 months to not reached）, respectively, after a median follow-up of 33.6（95％*CI* 17.4–38.8）months. Lymphopenia（74.4％）, neutropenia（52.6％）, and leukopenia（39.7％）, thrombocytopenia（29.5％）and anemia（15.4％）were the most common grade 3–4 hematologic adverse events（AE）. The most frequent non-hematologic AEs included nausea（43.6％）, vomiting（33.3％）, and anorexia（29.5％）. Univariate and multivariate analysis showed that <4 cycles of bendamustine treatment was a poor prognostic factor for PFS（*P*＝0.003）, and failure to accept fludarabine containing regimen was a poor prognostic factor for OS（*P*＝0.009）.

**Conclusion:**

Bendamustine monotherapy has good efficacy and safety in the treatment of patient with R/R B-NHL.

我国惰性B细胞非霍奇金淋巴瘤（iB-NHL）的发病率在非霍奇金淋巴瘤中占23.7％[1]，临床进展缓慢，晚期患者不可治愈，中位生存时间为10～20年[2]。套细胞淋巴瘤（MCL）在亚洲人群中的发病率为2％～6％[3]，侵袭性较强且预后较差[3]–[6]。

苯达莫司汀是一种细胞毒药物，同时具有氮芥类结构和苯丙咪唑环，因此可以发挥烷化剂和嘌呤类似物的双重抗肿瘤效应[7]。多项研究显示，苯达莫司汀单药治疗复发难治iB-NHL和MCL有较高的缓解率和较长的缓解持续时间[4],[8]–[11]。目前国内有关苯达莫司汀的相关研究较少。为了评估苯达莫司汀在中国复发难治iB-NHL和MCL患者中的有效性和安全性，我们开展了一项前瞻性、多中心、开放性Ⅱ期临床研究。

## 病例与方法

1. 病例：主要入组标准如下：①年龄20～75岁。②接受含或不含利妥昔单抗治疗后疾病进展或治疗时疾病未缓解的B细胞非霍奇金淋巴瘤患者。包括滤泡性淋巴瘤（FL）、MCL、边缘区淋巴瘤（MZL）、小淋巴细胞淋巴瘤（SLL）、淋巴浆细胞性淋巴瘤（LPL）等。难治疾病定义为末次系统性全身治疗未达部分缓解（PR）及以上疗效或距末次系统性全身治疗12个月内再次出现疾病进展；利妥昔单抗耐药定义为在利妥昔单抗治疗期间疾病未缓解或者出现疾病进展或者在末次利妥昔单抗治疗结束之后6个月内出现疾病进展。③患者入组前曾接受过1～3线化疗（含或不含利妥昔单抗）；至少有一个可测量病灶，最长径大于1.5 cm，短径大于1.0 cm；或外周血B淋巴细胞绝对值≥5.0×10^9^/L。④入组前14 d内血常规检查满足HGB≥80 g/L，中性粒细胞计数（ANC）≥1.0×10^9^/L，PLT≥75×10^9^/L。⑤肝肾功能满足：总胆红素（TBIL）≤1.5×正常值上限（ULN），丙氨酸转氨酶（ALT）、天冬氨酸转氨酶（AST）均≤2×ULN，肌酐（Cr）≤1.5×ULN。⑥美国东部肿瘤协作组（ECOG）评分0～2分，预计生存期≥3个月。⑦患者签署知情同意书。主要排除标准：①根据研究者判断，不能耐受苯达莫司汀治疗的患者；②诊断为3b级FL或已转化为弥漫大B细胞淋巴瘤（DLBCL）的iB-NHL；③入组前1个月内进行任何较大的手术治疗或规律服用大剂量糖皮质激素；④有中枢神经系统侵犯或中枢神经系统疾病史；⑤患有除淋巴瘤以外的其他类型的活动性肿瘤；⑥活动性或严重感染。

2. 治疗：非SLL患者接受苯达莫司汀单药120 mg·m^−2^·d^−1^治疗6～8个周期，每周期第1、2天给药，21 d为1个周期，直至疾病进展或患者出现不可耐受的毒性。SLL患者药物剂量为100 mg·m^−2^·d^−1^，第1、2天给药，28 d为1个周期，共6～8个周期。

3. 随访：按照方案随访计划，患者在随访窗口期内接受门诊、电话或住院随访。主要研究终点为研究者根据2014年恶性淋巴瘤疗效评价标准（Lugano标准）判定的客观缓解率（ORR），包括完全缓解（CR）率和部分缓解（PR）率。次要研究终点为缓解持续时间（DOR）、无进展生存（PFS）期、总生存（OS）期和安全性。PFS期定义为自患者入组至第一次疾病进展或任何原因导致死亡的时间，DOR定义为第一次影像学评估为获得客观缓解至第一次评估为疾病进展（PD）或PD前因任何原因死亡的时间；OS期定义为自患者入组至因任何原因死亡的时间。根据第四版《不良反应通用术语标准（CTCAE）》评估受试者的不良事件（AEs）。

4. 统计学处理：使用SAS 9.2软件进行统计学分析。所有统计学检验均采用双侧检验，*P*≤0.05为差异有统计学意义，可信区间采用95％*CI*。计数资料用例数（百分比）表示，计量资料用中位数（范围）或中位数（95％*CI*）表示。ORR为获得CR和PR患者所占比例。PFS、DOR和OS通过Kaplan-Meier法计算。全分析集（FAS集）定义为按照意向性分析（ITT）原则，对所有入组且至少应用1次药物的全部病例进行疗效分析。符合方案集（PP集）定义为完成2个及以上疗程、符合试验方案、依从性高、试验期间未应用禁止用药、完成病例报告表规定填写内容的病例。针对FAS和PP集分别计算有效率。安全性分析集（SS）为至少接受1次治疗并有安全性数据的患者。

## 结果

1. 临床特征：2012年3月至2016年12月，全国11所医院共入组78例患者，其中51例为iB-NHL，27例为MCL。患者基线情况见表1。男52例（66.7％），患者中位年龄58（24～76）岁，69例（88.4％）患者Ann Arbor分期Ⅲ～Ⅳ期。在iB-NHL患者中，FL患者24例（30.8％），SLL患者24例（30.8％），MZL患者2例（2.6％），LPL患者1例（1.3％）。患者既往均接受过化疗，48例（61.5％）患者既往接受过2线及以上治疗。52例（66.7％）患者既往接受过利妥昔单抗治疗，41例（52.6％）患者对利妥昔单抗耐药。74例（94.9％）患者接受过含烷化剂方案的治疗。患者末线治疗至入组本研究的中位时间为5.2个月。

**表1:** 78例复发/难治B细胞非霍奇金淋巴瘤患者的基线临床特征

特征	总体（78例）	iB-NHL（51例）	MCL（27例）
男性[例（%）]	52（66.7）	32（62.7）	20（74.1）
女性[例（%）]	26（33.3）	19（37.3）	7（25.9）
年龄[岁，*M*（范围）]	58（24～76）	56（24～76）	61（42～73）
对既往治疗的反应[例（%）]			
复发	7（9.0）	7（13.7）	0（0）
难治	61（78.2）	37（72.5）	24（88.9）
不确定	10（12.8）	7（13.7）	3（11.1）
距末次治疗的中位时间[月，*M*（范围）]	5.2（0.4～86.9）	3.0（0.4～34.7）	5.8（0.4～86.9）
ECOG评分[例（%）]		
0分	45（57.7）	34（66.7）	11（40.7）
1分	24（30.8）	13（25.5）	11（40.7）
2分	2（2.6）	0（0）	2（7.4）
不确定	7（9.0）	4（7.8）	3（11.1）
Ann Arbor分期[例（%）]			
Ⅰ	5（6.4）	3（5.8）	2（7.4）
Ⅱ	4（5.1）	3（5.8）	1（3.7）
Ⅲ	37（47.4）	26（51.0）	11（40.7）
Ⅳ	32（41.0）	19（37.3）	13（48.1）
病理类型[例（%）]			
MCL	27（34.6）	-	27（100.0）
FL	24（30.8）	24（47.1）	-
SLL	24（30.8）	24（47.1）	-
MZL	2（2.6）	2（3.9）	-
LPL	1（1.3）	1（2.0）	-
FLIPI评分[例（%）]^a^			
低危（0～1分）	2（8.3）	-	-
中危（2分）	7（29.2）	-	-
高危（>2分）	15（62.5）	-	-
MIPI评分[例（%）]			
低危（0～3分）	-	-	10（37.0）
中危（4～5分）	-	-	11（40.7）
高危（6～11分）	-	-	6（22.2）
是否侵犯骨髓[例（%）]			
是	15（19.2）	13（25.5）	2（7.4）
否	58（74.4）	37（72.5）	21（77.8）
不确定	5（6.4）	1（2.0）	4（14.8）
既往治疗线数[例（%）]		
1	30（38.5）	22（43.1）	8（29.6）
2	25（32.1）	16（31.4）	9（33.3）
3	11（14.1）	6（11.8）	5（18.5）
>3	12（15.4）	7（13.7）	5（18.5）
既往治疗方案[例（%）]			
含利妥昔单抗方案	52（66.7）	37（72.5）	15（55.6）
含氟达拉滨方案	34（43.6）	28（54.9）	6（22.2）
含烷化剂方案	74（94.9）	47（92.2）	27（100.0）

注 iB-NHL：惰性B细胞非霍奇金淋巴瘤；MCL：套细胞淋巴瘤；ECOG：美国东部肿瘤协作组；FL：滤泡性淋巴瘤；SLL：小淋巴细胞淋巴瘤；MZL：边缘区淋巴瘤；LPL：淋巴浆细胞性淋巴瘤；FLIPI：滤泡性淋巴瘤国际预后指数；MIPI：套细胞淋巴瘤国际预后指数；-：不适用。^a^共24例患者进行了FLIPI评分

2. 苯达莫司汀暴露情况：所有患者中位接受4（1～10）个疗程治疗。13例（16.7％）患者进行了药物减量，减量主要发生在第1～3个疗程，11例（14.1％）患者仅接受1个周期治疗，其中6例（7.7％）由于AEs，3例主动退出，2例出现疾病进展。共56例（71.8％）患者未完成6个周期治疗，未完成原因分别为：18例（23.1％）由于AEs；22例（28.2％）由于患者依从性原因退出治疗；9例（11.5％）由于出现影像学证实的疾病进展；3例（3.8％）由于研究者判断疾病出现进展；3例（3.8％）由于研究者判断疗效持续稳定无法继续获益；1例（1.3％）由于患者失访。既往接受过含氟达拉滨方案治疗的患者对苯达莫司汀耐受性较差，仅4例（15％）完成≥6个周期治疗。在未接受过氟达拉滨治疗的患者中，17例（44％）患者完成≥6个周期治疗。

3. 疗效：FAS集共纳入78例患者，其中可进行疗效评估的患者68例（87.2％）。PP集共纳入65例患者，其中63例（96.9％）患者可进行疗效评估。全组患者ORR为61.5（95％*CI* 49.8～72.3）％，CR率为5.1（95％*CI* 1.4～12.6）％；iB-NHL患者的ORR为56.9（95％*CI* 42.2～70.7）％，CR率为5.9（95％*CI* 1.4～12.6）％；MCL患者的ORR为70.4（95％*CI* 49.8～86.2）％，CR率为3.7（95％*CI* 0.1～19.0）％。独立评估委员会评估了51例患者，与研究者评估结果之间的一致性为92.2％。PP集中，所有患者、iB-NHL和MCL患者的ORR分别为70.8（95％*CI* 58.2～81.4）％、64.3（95％*CI* 48.0～78.4）％和82.6（95％*CI* 61.2～95.0）％，CR率分别为6.4％、7.1％和4.3％。

FAS集的患者中位随访33.6（95％*CI* 17.4～38.8）个月，全组、iB-NHL和MCL患者的中位PFS期分别为8.7（95％*CI* 6.7～13.2）个月、11.8（95％*CI* 5.9～18.2）个月和8.7（95％*CI* 4.1～10.4）个月（图1A）；中位DOR分别为8.3（95％*CI* 5.5～14.0）个月、10.2（95％*CI* 4.3～18.9）个月、6.5（95％*CI* 2.8～15.2）个月；中位OS期分别为25.5个月（95％*CI* 14.2个月～未达到），27.7个月（95％*CI* 14.2个月～未达到）和21.7个月（95％*CI* 9.5个月～未达到）。所有患者1年OS率为73.8（95％*CI* 61.1～82.9）％，iB-NHL患者1年OS率为77.5（95％*CI* 61.0～87.7）％，MCL患者1年OS率为67.2（95％*CI* 44.7～82.1）％（图1B）。

PP集的患者中位随访33.7（95％*CI* 16.4～38.8）个月，全组、iB-NHL和MCL患者的中位PFS期分别为8.3（95％*CI* 5.9～11.8）个月、8.1（95％*CI* 5.7～18.2）个月和8.7（95％*CI* 4.1～10.4）个月（图1C），中位DOR分别为6.9（95％*CI* 5.5～13.9）个月、6.9（95％*CI* 4.3～16.5）个月和6.5（95％*CI* 2.8～15.2）个月，中位OS期分别为25.5个月（95％*CI* 13.8个月～未达到）、25.5个月（95％*CI* 12.3个月～未达到）和23.4个月（95％*CI* 12.8个月～未达到），1年OS率分别为74.2（95％*CI* 60.2～84.0）％、72.5（95％*CI* 53.5～84.8）％和76.5（95％*CI* 52.3～89.6）％（图1D）。

**图1:**
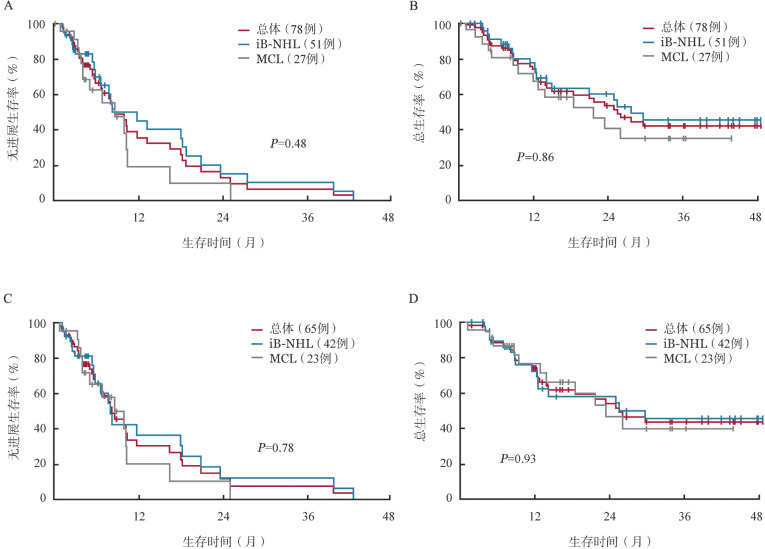
全分析集（A、B）和符合方案集（C、D）患者的无进展生存（PFS）和总生存（OS）曲线 注 iB-NHL：惰性B细胞非霍奇金淋巴瘤；MCL：套细胞淋巴瘤

根据病理亚型、性别、年龄、骨髓侵犯、LDH水平、疾病分期和既往治疗线数进行亚组分析，组间ORR差异无统计学意义。进一步进行单因素分析发现，接受苯达莫司汀≥4个周期患者的ORR优于<4个周期的患者［87.2（95％*CI* 72.6～95.7）％对46.2（95％*CI* 26.6～66.7）％，*P*<0.001］，其中接受苯达莫司汀治疗≥6个周期的患者ORR为100％。对以上影响因素进行PFS和OS的多因素分析，结果显示，接受苯达莫司汀治疗≥4个周期是PFS较好的影响因素（*HR*＝0.16，95％*CI* 0.05～0.49，*P*＝0.003），而既往接受含氟达拉滨方案是OS较好的影响因素（*HR*＝0.21，95％*CI* 0.07～0.65，*P*＝0.009）。

4. 安全性：最常见的3～4级血液学不良反应为淋巴细胞减少（74.4％）、中性粒细胞减少（52.6％）、白细胞减少（39.7％）、血小板减少（29.5％）和贫血（15.4％）。最常见的非血液学不良反应（不分级别，发生率>15％）为恶心（43.6％）、呕吐（33.3％）、厌食（29.5％）、体重减轻（23.1％）、发热（21.8％）、疲乏（20.5％）、丙氨酸转氨酶升高（20.5％）和天冬氨酸转氨酶升高（15.4％）。最常见的3～4级非血液学不良反应为肺部感染（9.0％）、上呼吸道感染（3.9％）和间质性肺病（3.9％）。研究过程中3例患者死亡，可能与苯达莫司汀用药相关，死亡原因分别为左心室射血分数减少、间质性肺病和肺部感染。

## 讨论

本研究为前瞻性、多中心Ⅱ期临床研究，旨在评估苯达莫司汀单药在中国复发/难治iB-NHL或MCL患者中的有效性和安全性。本研究患者的主要特征是88.4％为Ann Arbor分期Ⅲ/Ⅳ期，60％以上患者既往接受过≥2线化疗，提示该研究纳入患者大部分为疾病晚期且耐药性高。此外，62.5％的FL患者FLIPI评分为3～5分，高于既往Friedberg等[9]和Kahl等[8]研究所报道的比例，提示本研究入组的FL患者大部分预后不佳。全组患者中位年龄58岁，较欧美患者平均诊断年龄（MCL：68岁，NHL：67岁）低10岁左右[2],[12]。本研究的ORR和PFS与既往报道的苯达莫司汀在我国台湾和北美淋巴瘤患者中的结果相似[8],[13]，但低于日本的报道[4]，可能原因是日本的研究中高危风险患者比例更低（53.6％）。本研究中，FL和SLL患者的ORR分别为64％和61％，低于既往报道的数据（74％～90％和67％～71％）[4],[8]，可能是由于本研究中苯达莫司汀暴露水平较低（治疗周期少，更高比例患者减量）。目前，苯达莫司汀较少单药治疗MCL，多与利妥昔单抗或其他药物联合使用，ORR为63％～91％[14]–[16]。本研究纳入27例MCL患者，单药ORR即达到83％，在MCL中显示出积极的疗效，为苯达莫司汀单药治疗复发/难治MCL补充了循证学依据，未来将探索联合方案以进一步提高MCL疗效。

研究发现使用苯达莫司汀治疗≥4个周期患者的ORR和PFS率均显著高于<4个周期的患者。而苯达莫司汀治疗超过6个周期的ORR是100％。由此可见，疗效与苯达莫司汀疗程数存在相关性。因此，应尽量优化给药剂量，保证按时、足疗程给药。

本研究中苯达莫司汀的起始剂量为120 mg·m^−2^·d^−1^，SLL的起始剂量为100 mg·m^−2^·d^−1^，均是欧美国家标准剂量[4],[8]–[9]，但中国患者对此剂量可能耐受性不佳。本研究中主要不良反应表现为血液学不良反应和感染。本研究3～4级感染发生率为15.4％，其中3～4级肺部感染发生率为9％。尽管感染发生率与国外报道相近，但仍需关注苯达莫司汀导致免疫抑制的现象。Friedberg等[9]报道的3～4级淋巴细胞减少发生率为25％，而本组患者中3～4级淋巴细胞减少发生率为74.4％，因此可能有较高的感染发生率以及较长的免疫抑制时间。因此，结合国内各中心应用苯达莫司汀的临床经验，起始剂量调整为90 mg·m^−2^·d^−1^可能更加安全合理。

本研究显示，苯达莫司汀单药在中国iB-NHL和MCL患者中疗效确切，且耐受性良好。未来联合用药策略值得进一步研究探索。

## References

[1] 李 小秋, 李 甘地, 高 子芬 (2012). 中国淋巴瘤亚型分布: 国内多中心性病例10002例分析[J]. 诊断学理论与实践.

[2] Lumish M, Falchi L, Imber BS (2021). How we treat mature B-cell neoplasms (indolent B-cell lymphomas)[J]. J Hematol Oncol.

[3] Yoon DH, Cao J, Chen TY (2020). Treatment of mantle cell lymphoma in Asia: a consensus paper from the Asian Lymphoma Study Group[J]. J Hematol Oncol.

[4] Ohmachi K, Ando K, Ogura M (2010). Multicenter phase II study of bendamustine for relapsed or refractory indolent B-cell non-Hodgkin lymphoma and mantle cell lymphoma[J]. Cancer Sci.

[5] Teodorovic I, Pittaluga S, Kluin-Nelemans JC (1995). Efficacy of four different regimens in 64 mantle-cell lymphoma cases: clinicopathologic comparison with 498 other non-Hodgkin's lymphoma subtypes. European Organization for the Research and Treatment of Cancer Lymphoma Cooperative Group[J]. J Clin Oncol.

[6] Yatabe Y, Suzuki R, Tobinai K (2000). Significance of cyclin D1 overexpression for the diagnosis of mantle cell lymphoma: a clinicopathologic comparison of cyclin D1-positive MCL and cyclin D1-negative MCL-like B-cell lymphoma[J]. Blood.

[7] Cheson BD, Rummel MJ (2009). Bendamustine: rebirth of an old drug[J]. J Clin Oncol.

[8] Kahl BS, Bartlett NL, Leonard JP (2010). Bendamustine is effective therapy in patients with rituximab-refractory, indolent B-cell non-Hodgkin lymphoma: results from a Multicenter Study[J]. Cancer.

[9] Friedberg JW, Cohen P, Chen L (2008). Bendamustine in patients with rituximab-refractory indolent and transformed non-Hodgkin's lymphoma: results from a phase II multicenter, single-agent study[J]. J Clin Oncol.

[10] Cheson BD, Friedberg JW, Kahl BS (2010). Bendamustine produces durable responses with an acceptable safety profile in patients with rituximab-refractory indolent non-Hodgkin lymphoma[J]. Clin Lymphoma Myeloma Leuk.

[11] Vidal L, Gurion R, Shargian L (2019). Bendamustine for patients with indolent B cell lymphoproliferative malignancies including chronic lymphocytic leukaemia — an updated meta-analysis[J]. Br J Haematol.

[12] Zhou Y, Wang H, Fang W (2008). Incidence trends of mantle cell lymphoma in the United States between 1992 and 2004[J]. Cancer.

[13] Hsiao LT, Tien HF, Kuo CY (2015). Pharmacokinetic profile and first preliminary clinical evaluation of bendamustine in Taiwanese patients with heavily pretreated indolent B-cell non-Hodgkin lymphoma and mantle cell lymphoma[J]. Hematol Oncol.

[14] Visco C, Di Rocco A, Evangelista A (2021). Outcomes in first relapsed-refractory younger patients with mantle cell lymphoma: results from the MANTLE-FIRST study[J]. Leukemia.

[15] McCulloch R, Visco C, Eyre TA (2020). Efficacy of R-BAC in relapsed, refractory mantle cell lymphoma post BTK inhibitor therapy[J]. Br J Haematol.

[16] Hess G, Keller U, Scholz CW (2015). Safety and efficacy of Temsirolimus in combination with Bendamustine and Rituximab in relapsed mantle cell and follicular lymphoma[J]. Leukemia.

